# Clinical heterogeneity under induction with different dosages of cytarabine in core binding factor acute myeloid leukaemia

**DOI:** 10.1038/s41598-020-57414-y

**Published:** 2020-01-20

**Authors:** Biao Wang, Jihong Zhang, Xiaoying Hua, Haiqian Li, Zhilin Wang, Bin Yang

**Affiliations:** 1 Changzhou First People’s Hospital, Department of Hematology, Changzhou, 213000 China; 2 Shengjing Hospital of China Medical University, Blood Research Laboratory, Shenyang, 110000 China

**Keywords:** Cancer genomics, Chemotherapy, Acute myeloid leukaemia

## Abstract

Repeated cycles of post-remission high-dose cytarabine (Ara-C) have been suggested to improve survival in core binding factor (CBF) acute myeloid leukaemia (AML). High-dose Ara-C used for induction regimens has also been reported to be associated with increased treatment-related mortality (TRM). Few data are available about intermediate-dose Ara-C serving as induction therapy. The aim of our study was to compare the tolerance and outcomes of standard- and intermediate-dose levels of Ara-C as induction in CBF AML and to analyse the clinical heterogeneity of the two AML entities under these induction settings. We retrospectively investigated the outcomes in adults with CBF AML induced with regimens based on standard-dose Ara-C at 100 to 200 mg/m^2^ or intermediate-dose Ara-C at 1,000 mg/m^2^. In total, 152 patients with t(8; 21) and 54 patients with inv(16) AML were administered an induction regimen containing anthracyclines plus either standard- or intermediate-dose Ara-C. After a single course of induction, the complete remission (CR) rate in the inv(16) cohort was 52/52 (100%), higher than the 127/147 (86.4%) in the t(8; 21) cohort (*P* = 0.005). Intermediate-dose Ara-C (HR = 9.931 [2.135–46.188], *P* = 0.003) and negative *KIT*mut (HR = 0.304 [0.106–0.874], *P* = 0.027) independently produced an increased CR rate in the t(8; 21) cohort. Positive CD19 expression (HR = 0.133 [0.045–0.387], *P* = 0.000) and sex (male) (HR = 0.238 [0.085–0.667], *P* = 0.006) were associated with superior leukaemia-free survival (LFS) in the t(8; 21) cohort independently of *KIT*mut status or the induction regimen. We conclude that intermediate-dose Ara-C is superior to standard-dose Ara-C for induction of remission in t(8; 21) AML, and CD19 status and sex independently confer prognostic significance for LFS. The *KIT*mut status alone does not have an independent effect on survival in t(8; 21) AML. More intensive induction therapy is unnecessary in inv(16) AML.

## Introduction

Acute myeloid leukaemia (AML) with t(8; 21)(q22; q22) and AML with inv(16)(p13.1q22)/t(16; 16)(p13.1; q22) are together referred to as core binding factor (CBF) AML^1–4^, which constitutes approximately 5–8% of all *de novo* AML cases^5–7^. Both have been recognized as distinct diseases by the World Health Organization classification of myeloid neoplasms and acute leukaemia^8^.

Overall, CBF AML has a relatively favourable clinical outcome compared to other cytogenetic subtypes. Repeated cycles of high-dose cytarabine (Ara-C) for intensification during post-remission treatment can improve survival outcomes^9–12^. However, there is considerable clinicopathological heterogeneity within this AML subset, as demonstrated by the relapse incidence reaching up to 40% and the overall survival (OS) rate of 40–60%^13–17^.

Ara-C at a daily dose of 100 to 200 mg/m^2^ for 7 days as induction therapy is the most widely applied strategy in most centres. Several clinical trials evaluating high-dose Ara-C as induction therapy in AML have been conducted, with results differing and the majority reporting increased treatment-related toxicities^18–23^. Consensus has not been reached on the benefit of higher doses of Ara-C in the induction stage. A randomized trial showed no response and survival benefits but excessive toxic effects for high-dose Ara-C induction compared to intermediate-dose Ara-C induction in newly diagnosed AML patients aged 18 to 60 years, suggesting a plateau in the dose-response relationship above intermediate-dose Ara-C^20^. In view of this, the use of high-dose Ara-C for induction remains controversial.

However, few data are available regarding the impact of intermediate-dose Ara-C induction on clinical outcomes specifically in CBF AML. Additionally, in recent years, next-generation sequencing (NGS) technology has been widely used in AML. In this scenario, we conducted a comparison between standard- and intermediate-dose levels of Ara-C during induction in adults with CBF AML. Meanwhile, we analysed the impacts of clinicopathological characteristics (i.e., immunophenotyping, cytogenetics, and molecular biology data) and NGS-identified genetic lesions on clinical outcome under these induction settings.

## Results

### Patient baseline clinicopathological and genetic characteristics

In total, 206 patients with CBF AML consisting of 152 t(8; 21) and 54 inv(16) cases who fulfilled the enrolment criteria were included in the final analysis. The median age was 34 years (range 16–65 years), with male and female sex accounting for 107 (51.9%) and 99 (48.1%) patients, respectively. Supplementary Table S1 gives details on the data regarding patient clinicopathological and genetic characteristics at diagnosis according to CBF subtype.

### Induction therapy safety and response in CBF AML

The distribution of analysed patients was even in terms of all clinicopathological and genetic parameters between the two induction arms for both CBF subtypes (data not shown). The induction strategies were also balanced between both CBF cohorts, with the standard dose being given in 83 patients (54.6%) and the intermediate dose being given in 69 patients (45.4%) in the t(8; 21) cohort and the standard dose being given in 31 patients (57.4%) and the intermediate dose being given in 23 patients (42.6%) in the inv(16) cohort (*P* = 0.722). In the 152 t(8; 21) patients, five patients died early of septic shock or cerebral haemorrhage during or after induction, with two patients (2.4%) being in the standard-dose arm and three patients (4.3%) being in the intermediate-dose arm. In the 54 inv(16) patients, there were 2 early deaths, with one in each induction arm. There was no significant difference in early mortalities between the two arms (*P* = 0.772). The remaining 147 patients with t(8; 21) and 52 patients with inv(16) were evaluable for response assessment. The overall CR rate in the t(8; 21) cohort was 127/147 patients (86.4%), with 63/81 patients (77.8%) responding in the standard-dose arm and 64/66 patients (97.0%) responding in the intermediate-dose arm (*P* = 0.001). All 52 patients in the inv(16) cohort achieved an overall CR after a single course of induction. (Supplementary Table S2).

Compared with the standard-dose arm, the intermediate-dose arm showed similar incidences of grade 3 and 4 toxicities and no delayed neutrophil and platelet recoveries. Overall, non-haematological toxicities were mild and controllable, without any neurotoxicity documented.

### Analysis of factors impacting CR in t(8; 21) AML

After analysis with a univariate chi-square test, in the t(8; 21) cohort, patients with *KIT* mutations (*KIT*muts) had a lower overall CR rate than those without *KIT*muts (*P* = 0.025), and the codon change D816 was more associated with the significant difference than N822 (*P* = 0.024 for D816; *P* = 0.256 for N822). In the standard-dose arm, a trend existed but did not reach a statistical significance (*P* = 0.107). An elevated fusion transcript level ( > 150 copies/ABL copies) was found to have an inferior impact on the CR rate only in the standard-dose arm (*P* = 0.046), while this effect was not obvious in the intermediate-dose arm (*P* = 1.000). The intermediate-dose Ara-C induction regimen raised the overall CR rate by nearly 20% versus the standard-dose Ara-C regimen (97.0 *vs*. 77.8%, *P* = 0.001). (Supplementary Table S2).

Logistic regression analysis revealed that in the entire t(8; 21) cohort, the factors independently associated with a higher CR rate were intermediate-dose induction (HR = 9.931 [2.135–46.188], *P* = 0.003) and negative *KIT*mut status (HR = 0.304 [0.106–0.874], *P* = 0.027). The stratified analysis identified fusion transcript level and *KIT*mut status as factors independently impacting the CR rate in the standard-dose arm, while no factor was identified in the intermediate-dose arm. Moreover, both univariate chi-square and multivariate logistic regression analyses yielded a consistent and robust statistical significance for the impact of induction regimen on the CR rate (*P* < 0.01 for both analyses), suggesting its powerful predictive capability, as demonstrated by the considerably high hazard ratio (HR) value (HR = 9.931). (Table 1).

**Table 1:** Multivariate logistic analysis of the CR rate in the entire t(8; 21) cohort and in both treatment arms.

Factors	Good	Entire t(8; 21) cohort			SD arm			ID arm		
		χ2 *P*	HR (95% CI)	*P*#	χ2 *P*	HR (95% CI)	*P*#	χ2 *P*	HR (95% CI)	*P*#
Fusion transcript ratio*	<150	0.141	NA	NA	**0**.**046**	**0**.**339 (0**.**118–0**.**973)**	**0**.**044**	1.000	NA	NA
*KIT*	(−)	**0**.**025**	**0**.**304 (0**.**106–0**.**874)**	**0**.**027**	0.107	**0**.**081 (0**.**008–0**.**831)**	**0**.**034**	0.118	NA	NA
*KIT*-D816	(−)	**0**.**024**	NA	NA	0.151	NA	NA	0.256	NA	NA
*KIT*-N822	(−)	0.256	NA	NA	0.783	NA	NA	**0**.**036**	NA	NA
Induction regimen	ID	**0**.**001**	**9**.**931 (2**.**135–46**.**188)**	**0**.**003**	NA	NA	NA	NA	NA	NA

Notes: SD, standard-dose; ID, intermediate-dose; NA, not applicable; *P*#, significance according to the forward LR method; *tested as dichotomous categorical variable divided near its median value. Parameters showing statistical significance are highlighted in bold.

No statistical difference was observed regarding CR rate between t(8; 21) patients with 2 or more concomitant additional chromosomal abnormalities and those without, as well as patients between patients with and without *FLT3*-ITD mutations, which has been well documented in cytogenetically normal AML.

### Analysis of LFS and OS in t(8; 21) AML

**Figure 1:**
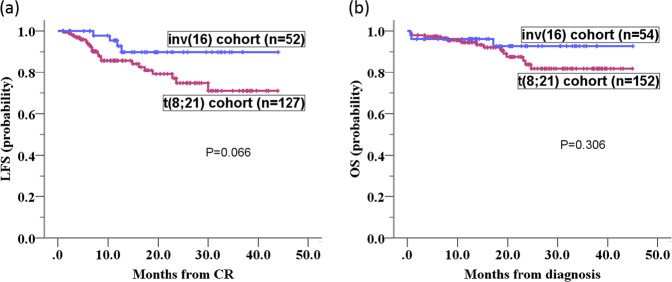
Kaplan-Meier plots of LFS and OS according to CBF subtype. (**a**) The inv(16) cohort showed a trend towards superior LFS compared to the t(8; 21) cohort, with a borderline significance (*P* = 0.066). (**b**) The OS between the two CBF cohorts was not statistically different (*P* = 0.306).

With a median follow-up of 12.7 (range 1–44) months, the inv(16) cohort showed a trend towards superior LFS versus the t(8; 21) cohort, but it did not reach statistical significance (*P* = 0.066). OS between the two cohorts showed no statistical difference (*P* = 0.306). (Fig. 1).

**Figure 2:**
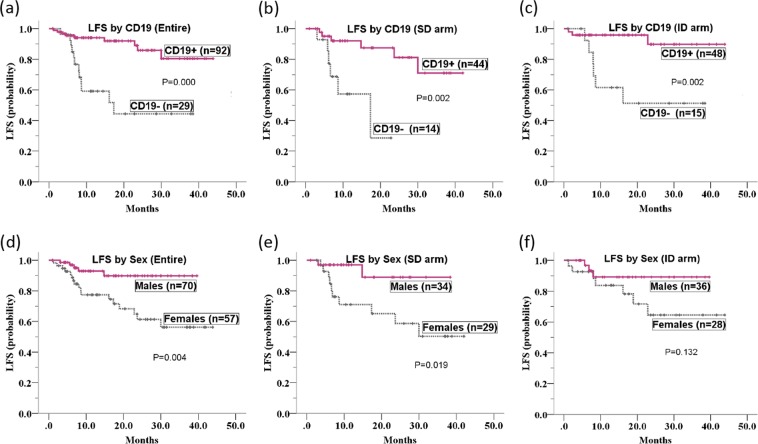
Kaplan-Meier plots of LFS according to CD19 expression or patient sex in the entire t(8; 21) cohort and in both treatment arms. (**a**–**c**) According to cohort- and induction-stratified outcome analysis, patients with negative CD19 expression exhibited poorer LFS than those with positive CD19 expression in the entire t(8; 21) cohort (**a**), the standard-dose Ara-C arm (**b**), and the intermediate-dose Ara-C arm (**c**) (*P* = 0.000, 0.002 and 0.002, respectively). (**d**,**e**) Female patients exhibited poorer LFS than male patients in both the entire t(8; 21) cohort (**d**) and the standard-dose arm (**e**) (*P* = 0.004 and 0.019, respectively). (**f**) Additionally, female patients showed slightly inferior LFS compared with male patients in the intermediate-dose arm, but the difference was not significant (*P* = 0.132).

In the t(8; 21) cohort, survival estimates were evaluable in 127 patients who attained CR1 and had received at least one consolidation cycle based on high-dose Ara-C. The median follow-up time was 11.4 months (range 1.1–43.9 months). In univariate Kaplan-Meier analysis, patients with positive *KIT*mut status and negative CD19 expression exhibited poorer LFS than those with negative *KIT*mut status and positive CD19 expression either in the entire t(8; 21) cohort, the standard-dose Ara-C arm, or the intermediate-dose Ara-C arm (*P* = 0.002, 0.021 and 0.037 for *KIT*, respectively; *P* = 0.000, 0.002 and 0.002 for CD19, respectively; Table 2 and Fig. 2). In the entire t(8; 21) cohort, male sex was shown to be associated with significantly superior LFS compared with female sex in the univariate Kaplan-Meier analysis (*P* = 0.006). Subgroup analysis indicated that this differences between male sex female sex was also present in the standard-dose arm (*P* = 0.019), while the LFS between males and females was not significantly different in the intermediate-dose arm (*P* = 0.132). When the screened factors were subjected to Cox regression models, the difference in LFS affected by CD19 and patient sex remained significant independently of *KIT*mut status in the entire t(8; 21) cohort and the standard-dose arm. Although *KIT*mut status was associated with inferior LFS in the entire t(8; 21) cohort and both induction arms according to the univariate Kaplan-Meier analysis, as was the 2 or more concomitant additional chromosomal abnormalities in standard-dose arm, these covariates were not confirmed in the multivariate Cox models. (Table 2).

**Table 2:** Multivariate Cox model of LFS in the entire t(8; 21) cohort and in both treatment arms.

Factors	Good	Kaplan-Meier analysis of LFS			Multivariate Cox regression analysis of LFS					
		*P* of entire cohort	*P* of SD arm	*P* of ID arm	HR (95% CI) of entire cohort	*P*	HR (95% CI) of SD arm	*P*	HR (95% CI) of ID arm	*P*
Sex	Male	**0**.**006**	**0**.**019**	0.132	**0**.**238 (0**.**085–0**.**667)**	**0**.**006**	**0**.**266 (0**.**055–1**.**276)**	**0**.**044**	NA	NA
CD19	(+)	**0**.**000**	**0**.**002**	**0**.**002**	**0**.**133 (0**.**045–0**.**387)**	**0**.**000**	**0**.**255 (0**.**058–1**.**111)**	**0**.**006**	**0**.**257 (0**.**056–1**.**175)**	**0**.**007**
≥2 ACAs	(−)	0.490	**0**.**009**	0.158	NA	NA	NA	NA	NA	NA
*KIT*	(−)	**0**.**002**	**0**.**021**	**0**.**037**	NA	NA	NA	NA	NA	NA
*NRAS*	(+)	0.069	0.181	0.216	NA	NA	NA	NA	NA	NA
*FLT3*-TKD	(−)	0.050	0.228	0.121	NA	NA	NA	NA	NA	NA
*NOTCH1*	(−)	0.089	0.515	**0**.**011**	**9**.**993 (1**.**888–52**.**900)**	**0**.**009**	NA	NA	NA	NA

Notes: SD, standard-dose; ID, intermediate-dose; NA: not applicable; ACAs, additional chromosomal abnormalities. Parameters showing statistical significance are highlighted in bold.

Univariate analysis demonstrated mutated *NRAS* to be associated with a superior LFS in the entire t(8; 21) cohort, with marginal significance (*P* = 0.069). Additionally, *FLT3*-TKD and *NOTCH1* mutations showed an association with poor LFS (*P* = 0.050 and 0.089, respectively), with *NOTCH1* showing independent significance in the multivariate model (HR = 9.993 [1.888–52.900], *P* = 0.009). (Table 2).

Because of the small number of patients who reached the study end-points, we were unable to perform multivariate Cox analysis for LFS in the inv(16) cohort.

### Combination of CD19 and sex can further refine risk stratification in t(8; 21) AML

**Figure 3:**
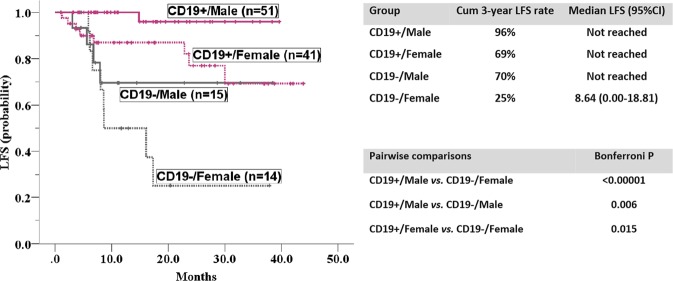
Kaplan-Meier plots of LFS according to CD19 expression combined with patient sex in the entire t(8; 21) cohort. CD19 expression in combination with patient sex led to a more obvious survival stratification for t(8; 21) AML, with male patients positive for CD19 expression having the most superior LFS and female patients negative for CD19 expression having the worst LFS (3-year LFS rate: 96% *vs*. 25%; median LFS duration: not reached *vs*. 8.6 months).

Given that CD19 and patient sex each showed an independent impact on LFS, we subsequently analysed their combined effect on clinical outcome in t(8; 21) AML. Given that there was no significant difference in LFS between the two induction arms, a Kaplan-Meier comparison was conducted collectively in the entire t(8; 21) cohort. When CD19 was combined with patient sex, the risk stratification was more obvious, with male patients with positive CD19 expression exhibiting the most superior LFS and female patients without CD19 expression showing the worst LFS (3-year LFS rate: 96% *vs*. 25%; median LFS duration: not reached *vs*. 8.6 months). (Fig. 3).

## Discussion

Although CBF AML has a relatively favourable prognosis compared with other non-M3 AML subtypes, there are yet heterogeneities across different CBF subtypes, making it possible to further improve the therapeutic effects in t(8; 21) AML patients. Dose-reduced induction chemotherapy has been suggested to predict a poor clinical outcome, as previously reported^24,25^, so our cohort of CBF patients were induced with adequate dose intensity and assessed for response after a single induction cycle. We identified the induction regimen and *KIT*mut status as the independent factors impacting the overall CR rate in the entire t(8; 21) cohort. The fusion transcript level and *KIT*mut status were independent factors in the standard-dose arm by subgroup analysis but not in the intermediate-dose arm. Patients who underwent intermediate-dose Ara-C induction had a nearly 20% increase in the overall CR rate with acceptable TRM similar to that of the standard-dose arm and could overcome the inferior influence of factors such as *KIT*mut status, which predict an impaired induction response. In addition, our data showed that there was no need to give more intensive Ara-C regimens as induction therapy for inv(16) patients, as indicated by the high CR rate as well as the low amount of patients who experienced relapse, irrespective of the dosage level.

Of the *KIT*muts, the codon change D816 was more associated with the significant difference in response than was N822 in t(8; 21) AML, a finding that is supported by *in vitro* studies demonstrating a positive association of the D816 mutation with stronger *KIT* phosphorylation and subsequent signalling activation^26,27^. In clinical practice, sequencing results are often not available fast enough, making the development of approaches that can quickly screen for the existence of *KIT*-D816 necessary. For instance, the ddPCR methodology can accomplish relatively quick evaluations, facilitating the timely administration of more intensive induction regimens in the distinctive *KIT*-D816-mutated t(8; 21) AML subset^28^.

No statistical difference was observed concerning the overall CR rate between t(8; 21) patients with 2 or more additional chromosomal abnormalities and those without, as well between patients with and without *FLT3*-ITD mutation. In the inv(16) cohort, the overall CR rate reached 100% regardless of the induction regimen, which is similar to the improved CR rates reported by others^29,30^.

Controversial prognostic values of *KIT*muts have been reported in CBF AML. Most studies confirmed *KIT*muts as predicting a higher risk of relapse and shorter OS^7,11,31–36^. In our t(8; 21) cohort, *KIT*muts showed a significant predictive value for LFS, with *KIT*-D816 mainly accountable for the difference compared to *KIT*-D822, consistent with previous reports^36^. However, the independent prognostic impact of *KIT*muts on LFS was lost in the multivariate model. We demonstrated that *KIT*muts alone, as a prognostic factor, did not confer an independently unfavourable impact on survival in t(8; 21) AML, which was similar to other results^37,38^. The possible explanations for the lack of findings of the effect of *KIT*muts on survival may be attributable to several explanations. (i) A low variant allele frequency (VAF) of *KIT*muts might be detected in a considerable percentage of patients owing to the relatively sensitive high-throughput NGS techniques. It has been shown that only *KIT*muts with a VAF of 25% or higher are independently correlated with an elevated relapse rate in t(8; 21) patients^39^. (ii) There is diversification of the clonal architecture as the leukaemia develops and progresses, as exemplified by the appearance of late evolutionary signalling clones. Mutations of *KIT*, *FLT3* and *RAS* have been commonly reported in CBF AML^32,40–42^. A recently published study described multiple signalling clones of *KIT*, *FLT3* and *RAS* variants during clonal progression^43^, which negatively affected event-free survival (EFS), while the presence of a single signalling clone showed no such relevance. iii) Post-remission high-dose Ara-C might still exert its role on residual *KIT*mut clones. iv) Mutations in *KIT* have also been reported to be frequently lost at disease relapse ^27,40^.

Congruent with data from multicentre studies of children in Germany^44^ and studies of French adults^39^, our findings showed mutated *NRAS* as being associated with favourable LFS, which is worth further attention and clarification. *NOTCH1* lesions commonly occur in more than 50% of T cell acute lymphoblastic leukaemia patients^45^. Activated Notch signalling contributes to the crosstalk between leukaemia cells and surrounding mesenchymal stromal cells, leading to malignant properties and chemoresistance^46,47^. We reported for the first time that mutated *NOTCH1* was an adverse prognostic factor in t(8; 21) AML. Due to the low incidence of mutated *NOTCH1* (10/152, 6.6%) in our t(8; 21) cohort, further confirmation of its clinical impact will be required.

The 3 + 7 combination scheme as induction therapy has still exerted its cornerstone role in modern therapies of AML. Because the anthracycline dosage in AML has reached its plateau (60–90 mg/m^2^), improved outcomes from induction with these 3 + 7 regimens depend on the dose adjustment of Ara-C. In an attempt to provide sufficient dose intensity, after failure on the first course of AML induction treatment, we prefer to change the drug combination scheme rather than to use the same combination for re-induction. Since a high dose (2,000–3,000 mg/m^2^ every 12 hours for 3 days or more) of Ara-C may increase the risk of induced toxicity or even early death, we did not include a high dose Ara-C in our AML induction strategy. In the current context, and also considering the tolerance of Chinese patients with AML, we designed an improved 3 + 7 regimen including intermediate-dose Ara-C at the late stage (days 5 to 7) of the induction course. Comparatively, the CR rates were compared after sequential double induction (i.e., two consecutive courses) from studies by Lowenberg *et al*.^20^, in which there was no significant difference in the remission rate between the two schemes. In addition, mutational data were not included in the outcome analysis of their relatively early investigations.

In our t(8; 21) cohort, although intermediate-dose Ara-C provided a remission advantage, the regimen as induction did not translate into an LFS benefit, which may be partly explained by repetitive cycles of post-remission high-dose Ara-C continuously preventing leukaemia recurrence. In line with our result, dose-intensified induction had no association with improved survival in the setting of post-remission intensification therapy in adult patients with CBF AML^11^.

Previous studies on the efficacy and outcome of CBF AML have rarely involved immunophenotypic data. A Japanese finding reported that CD19 status was significantly associated with CR rate (expression group 95.7% *vs*. non-expression group 83.8%, *P* = 0.049)^48^. However, we did not replicate this result either in the entire t(8; 21) cohort or in both induction arms. Intriguingly, in our study, negative CD19 expression and female sex presented a significantly higher relapse rate and a shorter LFS than positive CD19 expression and male sex, similar to a previous result^49^. CD19 expression remained a robust predictor of relapse in the multivariate Cox model, underscoring its independent prognostic implications. Furthermore, CD19 expression combined with sex can be used to further risk-stratify t(8; 21) patients, providing an unambiguous prognostication refinement for t(8; 21) AML.

There were some limitations in our study, including its non-prospective design and missing values owing to the unavailability of laboratory records, as well as patient loss to follow-up after referral to regional institutions. All of these factors can impair the power of the statistical comparisons. Prospectively randomized studies with larger sample sizes are warranted to redefine a high-risk subset from the relatively favourable but heterogeneous t(8; 21) AML populations.

Taken together, although sharing a common CBF lesion, these two AML subtypes display distinct clinical outcomes, with t(8; 21) AML showing relatively obvious heterogeneity. More intensive induction is unnecessary in inv(16) AML, where a trend towards slightly superior LFS is shown over t(8; 21) AML. Intermediate-dose Ara-C-containing induction may provide an optimized treatment option for t(8;21) patients, especially for those carrying elevated levels of fusion transcript and/or *KIT*muts, with acceptable tolerability and no increased early mortality. General *KIT*muts alone are not an independent factor for survival in t(8; 21) AML. CD19 expression and sex significantly affect LFS, and their combination can be used to risk-stratify t(8; 21) AML patients, thereby guiding individually risk-adapted post-remission therapy.

## Patients and Methods

### Patient selection

In the present study, we observed a cohort of 206 newly diagnosed CBF AML patients (aged 16 to 65 years) including 152 patients with t(8; 21)(q22; q22) [t(8; 21) patients] and 54 patients with inv(16)(p13q22)/t(16; 16)(p13; q22) [inv(16) patients] from August 2014 to March 2018. The study was conducted in accordance with the Declaration of Helsinki, and the protocol was approved by the Institutional Review Board of Changzhou First People’s Hospital and Shengjing Hospital of China Medical University. All patients provided signed informed consent for receiving therapies and using their records. For patients under 18 years of age, informed consent was also obtained from a parent and/or legal guardian. Patients who were eligible met the following requirements: t(8; 21) or inv(16) AML diagnoses established according to the WHO criteria^50^; age over 16 but less than 65 years; availability of records on chromosomal karyotype and NGS data; remission induction using a standard-dose or an intermediate-dose Ara-C-containing regimen; and at least one course of consolidation had been received for those achieving a complete remission (CR).

### Immunophenotyping by flow cytometry

Untreated bone marrow (BM) samples were freshly collected at first diagnosis. Four-colour immunophenotyping was performed by flow cytometry on a FACSCalibur instrument (Becton-Dickinson, CA, USA) using mouse antihuman monoclonal antibodies against the following myeloid and lymphoid associated markers labelled by combinatorial immunofluorescence FITC/PE/PerCP/APC sets: 1) CD34/CD10/CD45/CD19; 2) CD7/CD117/CD45/CD33; 3) CD9/CD2/CD45/CD56; 4) CD15/CD38/CD45/HLA-DR; 5) CD16/CD13/CD45/CD11b; 6) CD4/CD64/CD45/CD14; 7) cMPO/cCD79a/CD45/cCD3; and 8) TdT/CD123/CD45/HLA-DR. Cells were stained with different fluorescently labelled monoclonal antibodies according to the manufacturer’s protocol. For intracellular markers, a BD cytofix/cytoperm kit was used. Blasts were obtained and characterized based on combination gating of CD45/side scatter along with other antigen markers. The expression of lineage-specific markers was determined by analysis on a FACSCanto II or FACSAria (Becton-Dickinson). Results were expressed as the mean fluorescence intensity (MFI; arbitrary relative linear units, scaled from 0 to 10^4^), with negative expression defined at a fluorescence intensity of <10^2^, dim expression of 10^2^–10^3^ and strong expression of 10^3^–10^4^. Samples were considered positive if at least 20% of blasts had surface antigen expression.

### Conventional karyotyping and FISH analysis

Conventional metaphase karyotyping of untreated BM samples was performed using the G/R-banding method. Twenty metaphases were routinely counted in each patient, with karyotypes described according to the International System for Human Cytogenetic Nomenclature (ISCN)^51,52^. Interphase FISH was performed using a dual-colour, dual-fusion FISH (D-FISH) fluorescent probe specific for the chimeric genes *RUNX1*-*RUNX1T1* and *CBFβ*-*MYH11*. At least 500 cells per sample were analysed, and the number of cells showing abnormal fluorescence signals was calculated. The abnormal cut-off for the D-FISH probe sets was defined as > 0.6% of the 500 interphase cells analysed with the D-FISH probe set.

### Total RNA extraction and fusion transcript level measurement

Total RNA was extracted from mononuclear cells in fresh BM samples using TRIzol Reagent (Life Technologies, Grand Island, NY, USA). Fusion transcript levels were measured by real-time quantitative PCR (RQ-PCR) on a ViiA7 Dx PCR System (Applied Biosystems, Life Technologies, CA, USA) with a Leukemia-Related Fusion Gene Detection Kit.

### Next-generation sequencing (NGS)

Genomic DNA was extracted from fresh BM samples with the QIAamp DNA Mini Kit (Qiagen GmbH, Germany) following the manufacturer’s instructions. All cases with a sufficient amount of genomic DNA were subjected to comprehensive analysis of gene mutations with the Ion PGM™ or Illumina next-generation sequencer. The detection panel was comprised of 112 potentially mutated genes related to haematological malignancies involved in the following functional categories: epigenetic regulators, signalling pathways, transcription factors, spliceosomes, cohesin complex, tumour suppressors and *NPM1*. PCR followed by direct Sanger sequencing was used to detect *FLT3*-ITD, *NPM1*, *CEBPA* and other potential complex insertions and/or deletions as previously described^53–55^.

### Induction and consolidation treatment

Enrolled patients were administered either of the two regimens as induction chemotherapy: i) the standard-dose Ara-C arm consisted of Ara-C 100–200 mg/m^2^ i.v. over 24 hours from day 1 to 7 plus daunorubicin 60 mg/m^2^ or idarubicin 12 mg/m^2^ i.v. from day 1 to 3; ii) the intermediate-dose Ara-C arm consisted of Ara-C 1,000 mg/m^2^ i.v. over 2 hours every 12 hours from day 5 to 7, and other agents were the same as those in the standard-dose arm. BM response assessment was performed between days 21 and 28 after induction when peripheral blood (PB) counts recovered. When no PB recovery was noticed or leukaemic blasts persisted or reappeared in the PB, the response assessment was postponed to no later than day 35 after induction. Patients obtaining a first CR received consolidation chemotherapies based on intermediate- or high-dose Ara-C.

### Clinical end-points and definitions

The overall CR rate achieved after a single induction course, early mortality, leukaemia-free survival (LFS) and overall survival (OS) were evaluated. In this study, CR and complete remission with incomplete platelet or neutrophil recovery (CRi), which were established according to criteria^50^, were collectively termed the overall CR. Early mortality was defined as death within 30 days after induction before response assessment. Relapse was defined as the reappearance of 5% or more leukaemic blasts on BM aspirates, the presence of blasts at any percentage in the PB, or recurrence at an extramedullary site of disease for patients who had previously achieved overall CR. LFS was calculated as the interval from the date of overall CR documentation until either first relapse or death. OS was defined as the interval between diagnosis and death from any cause. Patients who underwent haematopoietic stem cell transplantation were censored from survival analysis on the date of transplantation.

### Statistical analysis

Median values (and ranges) were calculated for non-normally distributed data. The Mann-Whitney U test was used for analysis of nonparametric continuous variables. Frequencies were compared by the chi-square test for categorical variables after crosstabulation. Continuous variables were dichotomously transformed after subdivision near their median values. The probabilities of survival were estimated using the Kaplan-Meier method and were compared by the log-rank test between subgroups according to predictive factors. Comparisons were stratified by CBF subtype, clinicopathological parameters, and induction regimen. Factors that fulfilled the pre-specified assumption with *P*-values < 0.15 in univariate analyses were further examined in the multivariate model. Multivariate logistic regression analysis was used for factors impacting the achievement of CR and a multivariate Cox model was used for factors associated with survival end-points. All statistical tests were 2-sided, and *P*-values < 0.05 were considered statistically significant. Statistical analyses were conducted applying IBM SPSS Statistics 22 for Windows.

## Supplementary information

Supplementary Table S1. Patients’ baseline clinical and genetic features according to CBF subtype.

Supplementary Table S2. Univariate Chi-square test on CR rate in entire t(8;21) cohort and in both arms.

## Data Availability

The datasets generated during and/or analysed during the current study are available from the corresponding author on reasonable request.
