# Supplementary material for: Clinical heterogeneity under induction with different dosages of cytarabine in core binding factor acute myeloid leukaemia
Source: Sci Rep. 2020 Jan 20;10:685. doi: 10.1038/s41598-020-57414-y (PMC6971028; doi:10.1038/s41598-020-57414-y)
Supplement: Supplementary file 1 — Supplementary Table S1. Patients’ baseline clinical and genetic features according to CBF subtype. [file 41598_2020_57414_MOESM1_ESM.pdf]

SUPPLEMENTARY INFORMATION for article "**Clinical heterogeneity under induction with different dosages of cytarabine in core binding factor acute myeloid leukaemia**"

Biao Wang[1,+], Jihong Zhang[2,+], Xiaoying Hua[1], Haiqian Li[1], Zhilin Wang[1], and Bin Yang[1,*]

Supplementary Table S2. Univariate Chi-square test on CR rate in entire t(8;21) cohort and in both arms.

**Factors**	**levels**	**Entire cohort, n=147**		**SD arm, n=81**		**ID arm, n=66**		***P#***
		**CR rate, n/N (%)**	***P****	**CR rate, n/N (%)**	***P****	**CR rate, n/N (%)**	***P****	
Sex	Male	70/79 (88.6%)	0.203	34/43 (79.1%)	0.766	36/36 (100%)	0.399^{F}	***0.010***^{C}
	Female	57/68 (83.8%)		29/38 (76.3%)		28/30 (93.3%)		0.119^{C}
Age, y	<30	61/71 (85.9%)	0.870	29/38 (76.3%)	0.766	32/33 (97.0%)	1.000^{F}	***0.031***^{C}
	>30	66/76 (86.8%)		34/43 (79.1%)		32/33 (97.0%)		0.052^{C}
WBC count, $\times 10^9$/L	<10	71/82 (86.6%)	0.940	35/45 (77.8%)	1.000	36/37 (97.3%)	1.000^{F}	***0.024***^{C}
	>10	56/65 (86.2%)		28/36 (77.8%)		28/29 (96.6%)		0.069^{C}
Hb level, g/L	<80	73/87 (83.9%)	0.290	40/53 (75.5%)	0.492	33/34 (97.1%)	1.000^{F}	***0.008***
	>80	54/60 (90.0%)		23/28 (82.1%)		31/32 (96.9%)		0.143^{C}
PLT count, $\times 10^9$/L	<30	65/75 (86.7%)	0.922	36/44 (81.8%)	0.340	29/31 (93.5%)	0.217^{F}	0.260^{C}
	>30	62/72 (86.1%)		27/37 (73.0%)		35/35 (100%)		***0.003***^{C}
Fusion transcript level, %	<150	85/95 (89.5%)	0.141	47/56 (83.9%)	***0.046***	38/39 (97.4%)	1.000^{F}	0.077^{C}
	>150	42/52 (80.8%)		16/25 (64.0%)		26/27 (96.3%)		***0.009***^{C}
t(8;21) alone	yes	39/46 (84.8%)	0.700	20/26 (76.9%)	0.899	19/20 (95.0%)	0.517^{F}	0.201^{C}
	no	88/101 (87.1%)		43/55 (78.2%)		45/46 (97.8%)		***0.003***
Additional LOS	yes	67/75 (89.3%)	0.289	33/40 (82.5%)	0.313	34/35 (97.1%)	1.000^{F}	0.094^{C}
	no	60/72 (83.3%)		30/41 (73.2%)		30/31 (96.8%)		***0.008***
Additional del(9)	yes	6/8 (75.0%)	0.663^{C}	2/3 (66.7%)	0.535^{F}	4/5 (80.0%)	0.147^{F}	1.000^{F}
	no	121/139 (87.1%)		61/78 (78.2%)		60/61 (98.4%)		***0.000***
Additional +4	yes	4/5 (80.0%)	0.524^{F}	2/3 (66.7%)	0.535^{F}	2/2 (100%)	1.000^{F}	1.000^{F}
	no	123/142 (86.6%)		61/78 (78.2%)		62/64 (96.9%)		***0.001***
Additional +8	yes	3/3 (100%)	1.000^{F}	2/2 (100%)	1.000^{F}	1/1 (100%)	1.000^{F}	NA
	no	124/144 (86.1%)		61/79 (77.2%)		63/65 (96.9%)		***0.001***
Additional chr7 anomalies	yes	7/8 (87.5%)	1.000^{C}	1/2 (50.0%)	0.397^{F}	6/6 (100%)	1.000^{F}	0.250^{F}
	no	120/139 (86.3%)		62/79 (78.5%)		58/60 (96.7%)		***0.002***
Additional ≥2 karyotypes	yes	19/22 (86.4%)	1.000^{C}	9/11 (81.8%)	1.000^{F}	10/11 (90.9%)	0.308^{F}	1.000^{C}
	no	108/125 (86.4%)		54/70 (77.1%)		54/55 (98.2%)		***0.001***
KIT	(-)	81/88 (92.0%)	***0.025***	38/45 (84.4%)	0.107	43/43 (100%)	0.118^{F}	***0.021***^{C}
	(+)	46/59 (78.0%)		25/36 (69.4%)		21/23 (91.3%)		***0.048***
KIT-D816	(-)	107/119 (89.9%)	***0.024***^{C}	51/62 (82.3%)	0.151^{C}	56/57 (98.2%)	0.256^{F}	***0.004***
	(+)	20/28 (71.4%)		12/19 (63.2%)		8/9 (88.9%)		0.337^{C}
KIT-N822	(-)	106/120 (88.3%)	0.256^{C}	53/67 (79.1%)	0.783^{C}	53/53 (100%)	***0.036***^{F}	***0.000***
	(+)	21/27 (77.8%)		10/14 (71.4%)		11/13 (84.6%)		0.719^{C}
NRAS	(-)	108/125 (86.4%)	1.000^{C}	54/70 (77.1%)	1.000^{C}	54/55 (98.2%)	0.308^{F}	***0.001***
	(+)	19/22 (86.4%)		9/11 (81.8%)		10/11 (90.9%)		1.000^{C}
NRAS-G12	(-)	120/139 (86.3%)	1.000^{C}	59/77 (76.6%)	0.570^{F}	61/62 (98.4%)	0.118^{F}	***0.000***
	(+)	7/8 (87.5%)		4/4 (100%)		3/4 (75.0%)		1.000^{F}
NRAS-G13	(-)	117/136 (86.0%)	1.000^{C}	59/76 (77.6%)	1.000^{C}	58/60 (96.7%)	1.000^{F}	***0.001***
	(+)	10/11 (90.9%)		4/5 (80.0%)		6/6 (100%)		0.455^{F}
KRAS	(-)	121/141 (85.8%)	1.000^{F}	60/78 (76.9%)	1.000^{F}	61/63 (96.8%)	1.000^{F}	***0.001***
	(+)	6/6 (100%)		3/3 (100%)		3/3 (100%)		NA

FLT3	(-)	110/126 (87.3%)	0.659^{C}	55/70 (78.6%)	0.965^{C}	55/56 (98.2%)	0.282^{F}	***0.001***
	(+)	17/21 (81.0%)		8/11 (72.7%)		9/10 (90.0%)		0.652^{C}
FLT3-ITD	(-)	118/135 (87.4%)	0.446^{C}	58/74 (78.4%)	1.000^{C}	60/61 (98.4%)	0.147^{F}	***0.000***
	(+)	9/12 (75.0%)		5/7 (71.4%)		4/5 (80.0%)		1.000^{C}
FLT3 others	(-)	119/138 (86.2%)	1.000^{C}	60/77 (77.9%)	1.000^{F}	59/61 (96.7%)	1.000^{F}	***0.001***
	(+)	8/9 (88.9%)		3/4 (75.0%)		5/5 (100%)		0.444^{F}
CSF3R	(-)	114/130 (87.7%)	0.372^{C}	59/74 (79.7%)	0.369^{C}	55/56 (98.2%)	0.282^{F}	***0.001***
	(+)	13/17 (76.5%)		4/7 (57.1%)		9/10 (90%)		0.322^{C}
RELN	(-)	114/133 (85.7%)	0.740^{C}	57/74 (77.0%)	0.958^{C}	57/59 (96.6%)	1.000^{F}	***0.001***
	(+)	13/14 (92.9%)		6/7 (85.7%)		7/7 (100%)		1.000^{F}
JAK2	(-)	118/136 (86.8%)	0.998^{C}	59/75 (78.7%)	0.865^{C}	59/61 (96.7%)	1.000^{F}	***0.002***
	(+)	9/11 (81.8%)		4/6 (66.7%)		5/5 (100%)		0.455^{F}
NOTCH1	(-)	120/137 (87.6%)	0.276^{C}	58/73 (79.5%)	0.518^{C}	62/64 (96.9%)	1.000^{F}	***0.002***
	(+)	7/10 (70%)		5/8 (62.5%)		2/2 (100%)		1.000^{F}
NOTCH2	(-)	122/141 (86.5%)	0.591^{F}	60/78 (76.9%)	1.000^{F}	62/63 (98.4%)	0.090^{F}	***0.000***
	(+)	5/6 (83.3%)		3/3 (100%)		2/3 (66.7%)		1.000^{F}
SH2B3	(-)	121/138 (87.7%)	0.201^{C}	59/74 (79.7%)	0.369^{C}	62/64 (96.9%)	1.000^{F}	***0.002***
	(+)	6/9 (66.7%)		4/7 (57.1%)		2/2 (100%)		0.500^{F}
TET2	(-)	115/133 (86.5%)	1.000^{C}	59/75 (78.7%)	0.865^{C}	56/58 (96.6%)	1.000^{F}	***0.003***
	(+)	12/14 (85.7%)		4/6 (66.7%)		8/8 (100%)		0.165^{F}
KMT2D	(-)	116/134 (86.6%)	1.000^{C}	57/73 (78.1%)	1.000^{C}	59/61 (96.7%)	1.000^{F}	***0.002***
	(+)	11/13 (84.6%)		6/8 (75.0%)		5/5 (100%)		0.487^{F}
ASXL1	(-)	117/137 (85.4%)	0.411^{C}	58/76 (76.3%)	0.497^{C}	59/61 (96.7%)	1.000^{F}	***0.001***
	(+)	10/10 (100%)		5/5 (100%)		5/5 (100%)		NA
CREBBP	(-)	119/139 (85.6%)	0.533^{C}	58/76 (76.3%)	0.497^{C}	61/63 (96.8%)	1.000^{F}	***0.001***
	(+)	8/8 (100%)		5/5 (100%)		3/3 (100%)		NA
EP300	(-)	120/140 (85.7%)	0.594^{F}	59/77 (76.6%)	0.570^{F}	61.63 (96.8%)	1.000^{F}	***0.001***
	(+)	7/7 (100%)		4/4 (100%)		3/3 (100%)		NA
WT1	(-)	121/141 (85.8%)	1.000^{F}	61/79 (77.2%)	1.000^{F}	60/62 (96.8%)	1.000^{F}	***0.001***
	(+)	6/6 (100%)		2/2 (100%)		4/4 (100%)		NA
SETBP1	(-)	124/142 (87.3%)	0.137^{F}	62/78 (79.5%)	0.123^{F}	62/64 (96.9%)	1.000^{F}	***0.002***
	(+)	3/5 (60.0%)		1/3 (33.3%)		2/2 (100%)		0.400^{F}
CEBPA	(-)	125/145 (86.2%)	1.000^{F}	63/81 (77.8%)	NA	62/64 (96.9%)	1.000^{F}	***0.001***
	(+)	2/2 (100%)		0/0 (0%)		2/2 (100%)		NA
FAT1	(-)	110/128 (85.9%)	0.951^{C}	53/69 (76.8%)	0.900^{C}	57/59 (96.6%)	1.000^{F}	***0.001***
	(+)	17/19 (89.5%)		10/12 (83.3%)		7/7 (100%)		0.509^{F}
All Patients		127/147 (86.4%)	NA	63/81 (77.8%)	NA	64/66 (97.0%)	NA	***0.001***

Notes: Prior to the multivariate analysis, univariate Chi-square test preliminarily screens predictive factors impacting overall CR rate in entire t(8;21) cohort and in both induction arms. Comparisons are stratified on different levels by these factors or induction arm. By default, All *P*-values are from Pearson Chi-square results, otherwise denoted with “C” (Continuity correction) or “F” (Fisher' exact test) as appropriately; *P**, *P*-values between factors of different levels; *P*#, *P*-values between SD and ID Ara-C arms; SD, standard-dose; ID, intermediate-dose; NA, not applicable. Parameters showing statistical significance are highlighted in bold and italic.
