# Supplementary material for: Clinical heterogeneity under induction with different dosages of cytarabine in core binding factor acute myeloid leukaemia
Source: Sci Rep. 2020 Jan 20;10:685. doi: 10.1038/s41598-020-57414-y (PMC6971028; doi:10.1038/s41598-020-57414-y)
Supplement: Supplementary file 2 — Supplementary Table S2. Univariate Chi-square test on CR rate in entire t(8;21) cohort and in both arms. [file 41598_2020_57414_MOESM2_ESM.pdf]

SUPPLEMENTARY INFORMATION for article “**Clinical heterogeneity under induction with different dosages of cytarabine in core binding factor acute myeloid leukaemia**”

Biao Wang[1,+], Jihong Zhang[2,+], Xiaoying Hua[1], Haiqian Li[1], Zhilin Wang[1], and Bin Yang[1,*]

Supplementary Table S1. Patients' baseline clinical and genetic features according to CBF subtype.

**Variables**	**t(8;21) AML, n=152**	**inv(16) AML, n=54**	***P#***
Median age (range), y	32 (16-65)	41(16-63)	***0.015***
Sex (male:female), N	80:72	27:27	0.740P
Median WBC count (range), $\times10^9$/L	8.4 (0.9-155.0)	35.9 (1.6-156.0)	***0.000***
Median Hb level (range), g/dL	75 (39-145)	86 (40-124)	0.064
Median PLT count (range), $\times10^9$/L	30 (2-195)	26 (3-121)	0.821
Median fusion transcript ratio (range), %	140.9 (18.9-817.6)	203.4 (64.4-551.8)	***0.000***
Immunophenotype, n/N (%)*			
CD34	145/146 (99.3%)	51/51 (100%)	1.000F
TdT	36/145 (24.8%)	6/49 (12.2%)	0.064P
HLA-DR	143/146 (97.9%)	48/52 (92.3%)	0.146C
CD117	146/146 (100%)	52/52 (100%)	NA
CD13	137/146 (93.8%)	51/52 (98.1%)	0.406C
CD33	144/145 (99.3%)	52/52 (100%)	1.000F
CD123	142/145 (97.9%)	52/52 (100%)	0.567F
CD38	134/140 (95.7%)	44/51 (86.3%)	***0.049C***
CD64	9/123 (7.3%)	12/43 (27.9%)	***0.000P***
CD56	112/131 (85.5%)	1/39 (2.6%)	***0.000P***
MPO	137/140 (97.9%)	43/50 (86.0%)	***0.004C***
CD9	47/106 (44.3%)	44/44 (100%)	***0.000P***
CD19	108/145 (74.5%)	1/49 (2.0%)	***0.000P***
CD79a	26/144 (18.1%)	1/49 (2.0%)	***0.000P***
CD7	15/134 (11.2%)	0/48 (0.0%)	***0.035C***
Cytogenetics, n/N (%)			
Translocation alone	48/152 (31.6%)	35/54 (64.8%)	***0.000P***
Additional LOS	77/152 (50.7%)	1/54 (1.9%)	***0.000P***
Additional del(9q)	9/152 (5.9%)	0/54 (0%)	0.150C
Additional +4	5/152 (3.3%)	0/54 (0%)	0.404C
Additional +8	3/152 (2.0%)	5/54 (9.3%)	***0.049C***
Additional +22	0/152 (0%)	13/54 (24.1%)	***0.000C***
Additional 7 abnormalities	8/152 (5.3%)	2/54 (3.7%)	0.929C
Additional ≥ 2 other abnormalities	22/152 (14.5%)	8/54 (14.8%)	0.951P
Three-way translocations	9/152 (5.9%)	0/54 (0%)	0.150C
Gene mutations, n/N (%)			
Signaling pathways			
KIT	62/152 (40.8%)	17/54 (31.5%)	0.227P
KIT-D816	31/152 (20.4%)	9/54 (16.7%)	0.552P
KIT-N822	28/152 (18.4%)	4/54 (7.4%)	0.055P
NRAS	22/152 (14.5%)	29/54 (53.7%)	***0.000P***
KRAS	6/152 (3.9%)	15/54 (27.8%)	***0.000P***
FLT3	22/152 (14.5%)	10/54 (18.5%)	0.481P
FLT3 ITD	12/152 (7.9%)	0/54 (0%)	0.074C
FLT3 others	10/152 (6.6%)	10/54 (18.5%)	***0.011P***

CSF3R	18/152 (11.8%)	1/54 (1.9%)	0.057C
RELN	14/152 (9.2%)	3/54 (5.6%)	0.582C
JAK2	12/152 (7.9%)	2/54 (3.7%)	0.461C
NOTCH1	10/152 (6.6%)	4/54 (7.4%)	1.000C
NOTCH2	6/152 (3.9%)	4/54 (7.4%)	0.517C
SH2B3	9/152 (5.9%)	1/54 (1.9%)	0.408C
Epigenetic regulators			
TET2	14/152 (9.2%)	4/54 (7.4%)	0.902C
KMT2D	14/152 (9.2%)	6/54 (11.1%)	0.685C
ASXL1	10/152 (6.6%)	3/54 (5.6%)	1.000C
CREBBP	8/152 (5.3%)	1/54 (1.9%)	0.505C
EP300	8/152 (5.3%)	4/54 (7.4%)	0.811C
Tumor suppressors			
FAT1	21/152 (13.8%)	11/54 (20.4%)	0.253P
WT1	6/152 (3.9%)	5/54 (9.3%)	0.255C
Transcription factors			
SETBP1	5/152 (3.3%)	5/54 (9.3%)	0.166C
CEBPA	3/152 (2.0%)	4/54 (7.4%)	0.145C

Notes: WBC, white blood cell; Hb, hemoglobin; PLT, platelet; LOS, loss of sex; P, pearson Chi-square; C, continuity correction; F, fisher' exact test; NA, not applicable; *, percentage according to an available data. *P*#, *P*-values were obtained from Chi-square test after crosstabulation for categorical variables and Mann-Whitney U test for continuous variables of non-normal distribution. Parameters showing statistical significance are highlighted in bold and italic.
